# Endovascular innovation in a hostile abdomen: Repairing aortic rupture with a physician-modified endograft

**DOI:** 10.1016/j.jvscit.2026.102263

**Published:** 2026-04-14

**Authors:** Jocelynne T. Dorotan, Nielsen Gabriel, April Hou, Rita Akumuo, Kenny Oh, R. Gregory Conway

**Affiliations:** Division of Vascular Surgery, Department of Surgery, Lewis Katz School of Medicine at Temple University, Philadelphia, PA

**Keywords:** Complex endovascular aortic repair, Physician-modified endograft, Hostile abdomen

## Abstract

Emergent endovascular aneurysm repair is challenging in cases with unfavorable anatomy. We describe a patient with prior small bowel transplants dependent on a hypertrophic inferior mesenteric artery and a right common iliac to superior mesenteric artery bypass presenting with contained infrarenal aortic rupture. Emergent repair used two physician-modified endografts: a Gore Excluder C3 with a fenestration for the inferior mesenteric artery and an excluder limb with a single fenestration for the iliac to superior mesenteric artery bypass. Both fenestrations were stented, with postoperative imaging confirming patency. This case highlights the adaptability of physician-modified endografts, emphasizing the value of individualized device planning.

Endovascular aneurysm repair (EVAR) has become a mainstay of treatment for infrarenal abdominal aortic aneurysms (AAAs) because it is associated with significantly lower perioperative mortality and morbidity compared with open surgical repair.^1^ However, up to 30% of infrarenal AAAs do not fulfil the anatomical criteria for off-the-shelf EVAR, which include short necks, severe angulation, involvement of visceral branches, and atypical vascular anatomy.^2^ During standard EVAR, the inferior mesenteric artery (IMA) is typically sacrificed, relying on the superior mesenteric artery (SMA) and bilateral internal iliac arteries for collateral flow. However, in patients with prior visceral arterial bypasses (eg, iliac-to-SMA bypass), SMA occlusion with compensatory IMA collateralization or prior colonic resection with patent IMA, preservation of visceral branches may provide critical perfusion. Consequently, patients excluded from EVAR may require open surgical repair or advanced endovascular techniques, including fenestrated or branched EVAR to treat the aneurysm.

We present a case of a 43-year-old man with a prior gunshot wound and small bowel transplantation whose native bowel was perfused by the IMA and donor bowel was perfused by a right common iliac artery (CIA)-donor-SMA bypass presenting with a ruptured infrarenal AAA that was successfully treated with a physician-modified endograft (PMEG). By using a PMEG to preserve multiple mesenteric inflow sources, this case demonstrates the versatility of endograft modification by providing an endovascular solution to cases with significant anatomical challenges. Written consent was obtained for this case report.

## Case report

**Fig 1:**
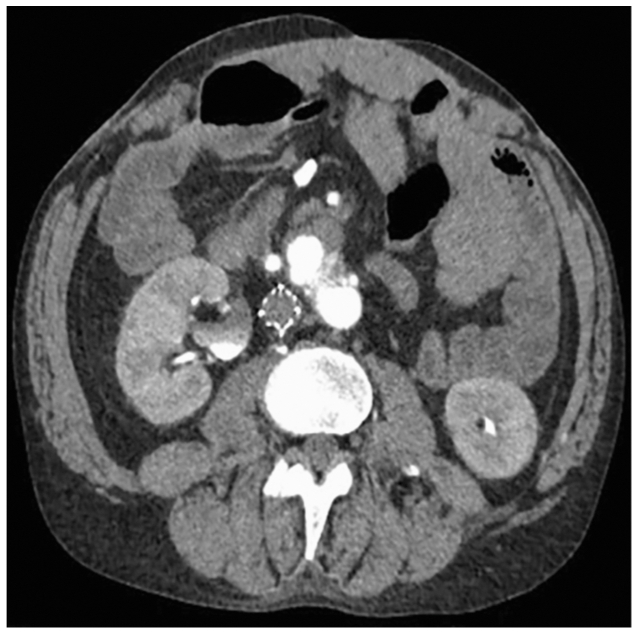
Computed tomography (CT) angiogram of the abdomen and pelvis before intervention.

The patient is a 43-year-old man with hypertension and a prior gunshot wound at age 23 complicated by short gut syndrome, for which he underwent small bowel transplantation at age 24 and retransplantation at age 30 for rejection. He is maintained on sirolimus and prednisone. Records from the initial trauma and transplant procedures were unavailable. He presented with a 3-day history of bright red blood per rectum, fatigue, and central abdominal pain. Initial evaluation revealed hemodynamic stability despite a hemoglobin of 4.0 g/dL, prompting transfusion of 2 U packed red blood cells while maintaining permissive hypotension. A computed tomography (CT) angiogram was significant for an infrarenal AAA measuring 4.2 × 2.5 cm, increased from 3.1 cm 5 years prior, with surrounding blood products (Fig 1). Although a mycotic etiology was considered given the patient's immunosuppressed state and transplant history, this was considered less likely given the absence of fever, normal white blood cell count (8-9 × 10^9^/L), and lack of radiographic features such as periaortic gas or inflammatory soft tissue changes. Although positron emission tomography with CT could further evaluate for infection, the contained rupture and hostile abdomen necessitated urgent repair, recognizing that an endovascular approach could serve as a temporizing strategy if infection were later identified. Based on the scan, his native bowel perfusion appeared to be dependent on a hypertrophied IMA and the donor bowel relied on a CIA-donor-SMA bypass. Owing to anatomical constraints and perfusion dependencies, he was unsuitable for any off-the-shelf endovascular devices for aneurysm repair. Additionally, extensive prior surgeries placed him at an increased risk for morbidity with an open approach. Considering these factors, we proceeded with an EVAR using a PMEG with stenting of the IMA and SMA bypass.

**Fig 2:**
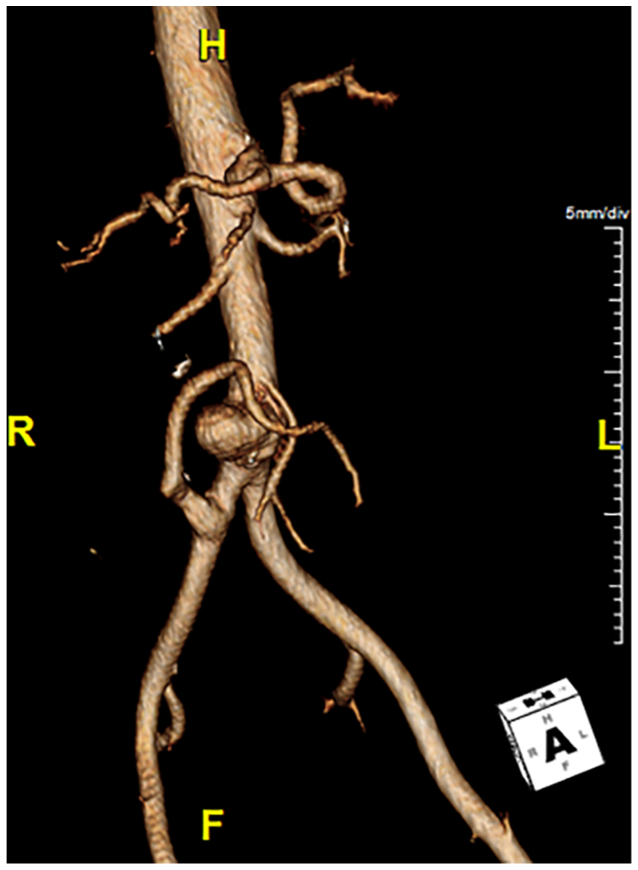
Three-dimensional reconstruction demonstrating aortic aneurysm anatomy and branch vessel orientation.

**Fig 3:**
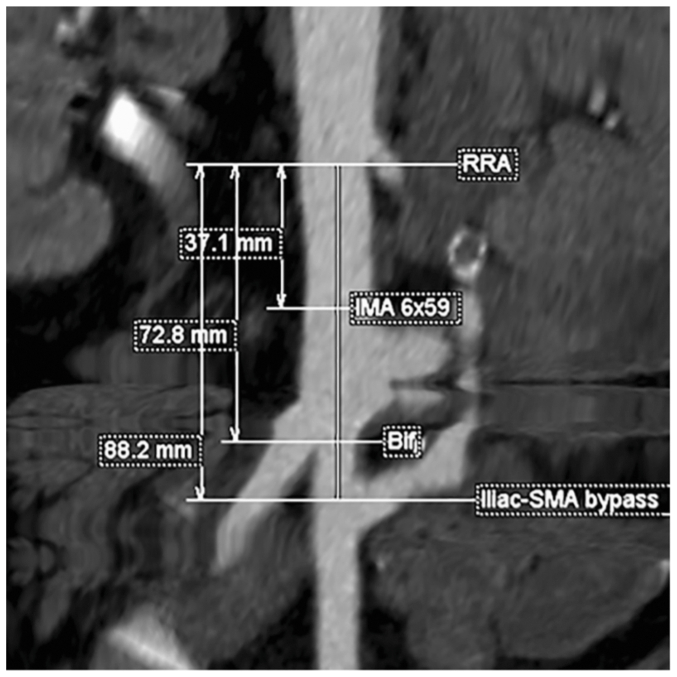
Preoperative computed tomography (CT) angiogram centerline measurements illustrating aneurysm anatomy and target vessel takeoffs. *Bif*, aortic bifurcation; *IMA*, inferior mesenteric artery; *RRA*, right renal artery; *SMA*, superior mesenteric artery.

The CT angiogram was evaluated with centerline imaging using Aquarius iNtuition Viewer (V4.7.1.16-59, TeraRecon Inc) (Fig 2). On centerline, there was 37.1 mm from the distal edge of the lowest right renal artery to the center of the IMA, 72.8 mm to the bifurcation, and 88.2 mm to the CIA-donor-SMA bypass. The IMA measured 6.4 mm in diameter. Given a distance of 72.8 mm from the lowest renal artery to the aortic bifurcation, a Gore Excluder C3 was selected, because it was the only device among available options that allowed the contralateral gate to open above the bifurcation (proximal fabric to gate distances: Cook Zenith, 82 mm; Medtronic Endurant II, 74 mm; Terumo Treo, 80 mm; Gore C3, 70 mm) (Fig 3). To accommodate the CIA-donor-SMA bypass, we chose to separately modify a 12 × 10 cm Gore Excluder iliac limb. Separately modifying two endografts was necessary for two reasons: precise clock-face and relative distance measurement accuracy was thought to be decreased by the transitioning of the centerline from the aorta to the iliac artery, and cannulation of the CIA-donor-SMA bypass would not be possible with the C3 delivery system in place. Modifying two separate devices allows the position to be more readily adjusted during implantation and for cannulation of the CIA-donor-SMA bypass fenestration before complete deployment of the modified iliac limb.

**Fig 4:**
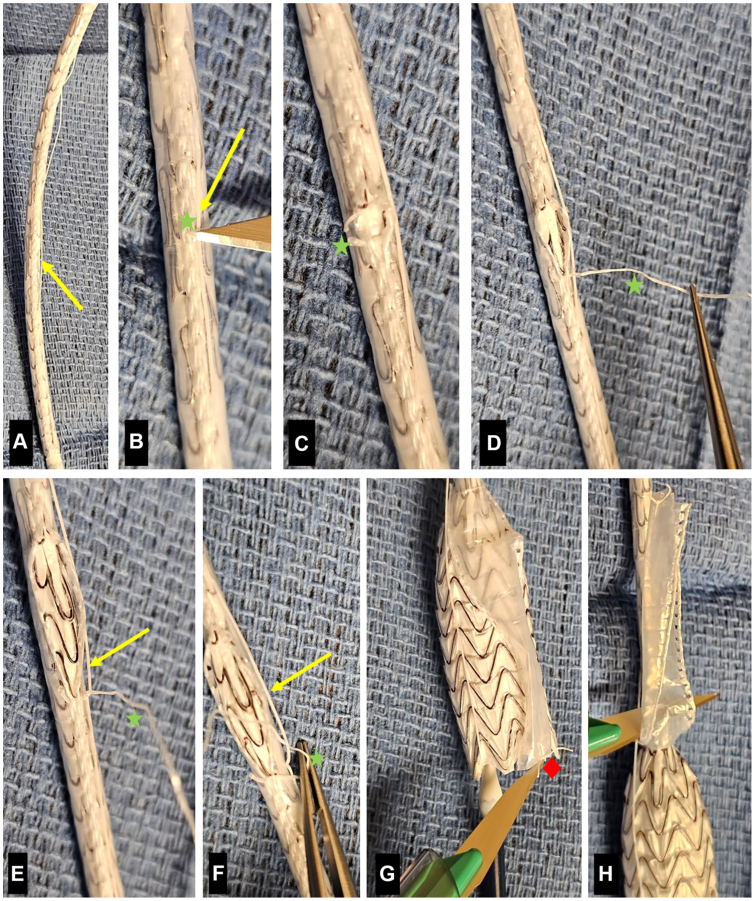
Exposure of the distal segment of the iliac limb. All images oriented with catheter tip at top. **(A)** Identify deployment suture (*yellow arrow*), which runs axially adjacent to the suture line of the containment membrane. **(B)** While protecting deployment suture, cut containment membrane sutures (*green star*). **(C)** Identify the cut end of the containment membrane suture. **(D)** Pull the containment membrane suture toward the distal end of device. **(E)** The containment membrane suture will cross the deployment suture. **(F)** Pass the containment suture under the loop of deployment suture. **(G)** Divide the distal containment membrane attachment suture (*red diamond*). **(H)** Reflect the containment membrane proximally and divide the excess.

When modifying the nonconformable 23-mm Gore C3, the outer constraining membrane was focally excised from the endograft at the appropriate location, and a 6-mm wire-reinforced fenestration was created. This fenestration was positioned just above the flow divider, in alignment with the IMA. The fenestration was reinforced with the coiled tip of a micropuncture wire, and the device was resheathed into an 18F sheath for delivery. The Gore Excluder iliac limb was modified using a single 8-mm wire-reinforced fenestration to align with the CIA-donor-SMA bypass. The outer constraining membrane was opened from the level of the planned fenestration to the distal end of the limb, allowing for controlled unsheathing and easier target vessel cannulation (Fig 4). The limb was resheathed into a 12F peel-away sheath for delivery.

**Fig 5:**
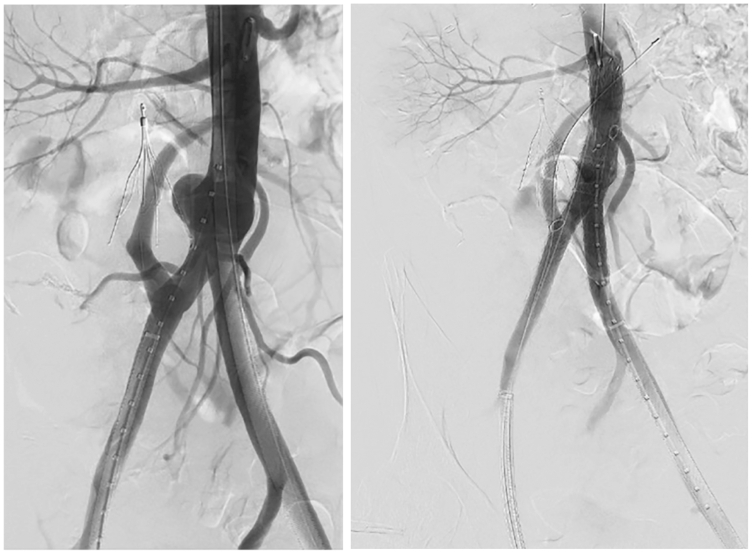
Angiogram before endovascular aneurysm repair (EVAR) demonstrating contained rupture, and presence of hypertrophic inferior mesenteric artery (IMA) and right iliac-superior mesenteric artery (SMA) bypass **(A)** and completion **(B)**.

The modified C3 was delivered through the left femoral access, positioned at the appropriate deployment marks, and deployed. The contralateral gate was cannulated from the right femoral access, and the IMA fenestration was selected with a steerable sheath (Tourguide, Aptus Medical, Medtronic). A 6 mm × 39 mm Gore VBX stent was positioned through the fenestration, and the C3 was completely deployed and ballooned with a Gore Molding and Occlusion Balloon. The VBX was then deployed and flared. Next, the modified limb was delivered through the right femoral access. The fenestration was aligned with the CIA-donor-SMA bypass, and the modified iliac limb was unsheathed. Because the constraining membrane was removed from the distal aspect of the iliac limb, the distal aspect of the iliac limb was already opened within the sheath. This maneuver facilitated easy wire cannulation by double-accessing the delivery sheath. Wire position within the CIA-donor-SMA was confirmed with contrast angiography, the iliac limb was completely deployed and was then ballooned with a Molding and Occlusion Balloon. An 8-mm Gore VBX stent was deployed into the CIA-donor-SMA and flared proximally (Fig 5). Completion angiography demonstrated exclusion of the aneurysm and patent IMA and CIA-donor-SMA bypass without endoleak.

**Fig 6:**
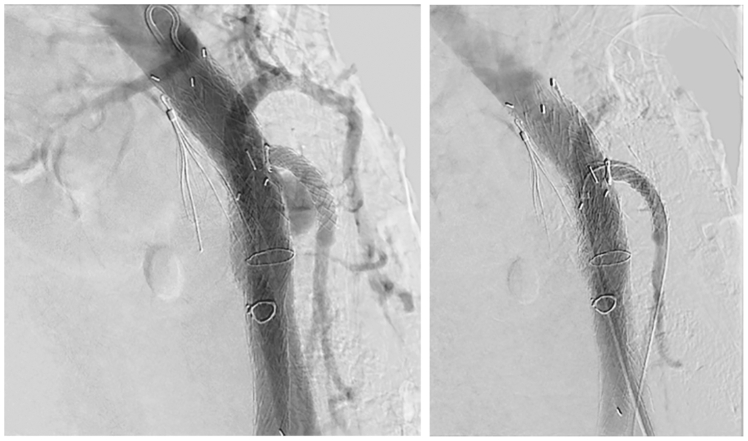
Angiogram showing type III endoleak **(A)** and resolution with relining **(B)** of the inferior mesenteric artery (IMA) stent.

The patient was briefly transferred to the surgical intensive care unit postoperatively for close monitoring, and then transferred to the surgical floor on postoperative day 1. The patient was discharged home on postoperative day 3. There were no complications at the patient's follow up appointment 6 weeks after the procedure. At 8 weeks after the procedure, however, the patient re-presented to the emergency department with rectal bleeding. Repeat angiography found a type III endoleak originating from the IMA fenestration. A 7 mm × 19 mm VBX stent was placed in the IMA, which resolved the endoleak. After this intervention, the IMA stent and the CIA-donor-SMA bypass remained patent (Fig 6). The patient was discharged home with no further complications. At follow-up appointment via telemedicine, he reported baseline functional status without recurrent bleeding or abdominal pain. He is scheduled for surveillance CT angiography.

## Discussion

EVAR remains the preferred approach for infrarenal AAA repair in appropriately selected patients owing to its reduced perioperative morbidity and mortality compared with open repair. Because anatomical criteria can exclude up to 30% to 40% of patients, advanced endovascular solutions like fenestrated-EVAR or branched EVAR grafts have been increasingly used.^3^ However, construction of these custom devices often requires 6 to 8 weeks, and they cannot be used in acute settings. Instead, PMEGs can be used to extend the applicability of EVAR to anatomically complex aortic pathology in acute settings, such as rupture.^4^ Alternative strategies, including chimney stenting of the IMA and a periscope graft to the right CIA-donor-SMA bypass with CERAB, were considered. However, we were concerned about the risk of proximal gutter-related endoleak and the limited caliber of the right CIA, which would not safely accommodate multiple parallel grafts without compromising seal integrity. Furthermore, open repair of this patient's contained infrarenal rupture would have required redo laparotomy with extensive lysis of adhesions followed by in situ aortic reconstruction with a prosthetic or cryopreserved conduit, reimplantation of the IMA, and reimplantation of the right CIA-donor-SMA bypass. An alternative strategy could include staged extra-anatomical axillobifemoral bypass with subsequent aortic and iliac ligation and IMA revascularization. Given the hostile abdomen, immunosuppression, and presence of transplanted bowel, these options were considered prohibitively high risk compared with a complex endovascular approach.

This case illustrates the application of a dual-fenestrated PMEG to preserve both the IMA and a CIA-donor-SMA bypass, highlighting the versatility of advanced endovascular approaches for complex aortomesenteric anatomy. Although conventional EVAR generally covers the IMA, as studies demonstrate no benefit to preservation owing to collateral supply, its maintenance was essential here.^5^^,^^6^ The use of two individually modified devices provided a tailored solution to the patient's unique vascular anatomy. Postoperatively, the patient presented with rectal bleeding, and imaging found a type III endoleak from the IMA fenestration. We believe this was likely due to inadequate flaring of the IMA bridging stent at the index procedure rather than a mismatch between fenestration and stent diameter. Colonoscopy demonstrated a normal ileocolonic anastomosis without evidence of ischemia or ulceration, and small bowel biopsy revealed chronic inflammatory changes without cytomegalovirus or dysplasia, suggesting that the bleeding was likely secondary to a primary gastrointestinal etiology and unrelated to the aneurysm repair. Type III endoleaks after PMEG repair occur in 13% to 23% of cases, more frequently with increased device modularity and branch incorporation; recurrence is reported in 15% to 5% within 2 years.^7^, ^8^, ^9^ Although this does not mandate intensified surveillance beyond standard EVAR protocols, strict lifelong imaging follow-up is essential.

Thus, this case illustrates that PMEGs offer a rapid, adaptable, and lifesaving option for emergent repair in anatomically complex scenarios where both open and standard EVAR approaches are unsuitable. Although advances in custom-made devices and off-the-shelf devices promise to treat a greater proportion of patients in accordance with manufacturer instructions for use, PMEGs will continue to have a role in treating patients with rare anatomical variants.

## Funding

None.

## Disclosures

None.
